# Persistent activation of growth hormone contributes to sexual dimorphic expression of hepatic drug metabolizing enzymes in *Fgf15* transgenic mice

**DOI:** 10.1016/j.dmd.2026.100368

**Published:** 2026-07-15

**Authors:** Manyun Dai, Hong Shi, Veronia Basaly, Vik Meadows, Bo Kong, Grace L. Guo

**Affiliations:** 1 Department of Pharmacology and Toxicology, Ernest Mario School of Pharmacy, Rutgers University, Piscataway, New Jersey; 2 Department of Public Health and Toxicology, School of Public Health, Health Science Center, Ningbo University, Ningbo, Zhejiang, China; 3 Department of Infectious Disease, Third Affiliated Hospital of Sun Yat-sen University, Guangzhou, Guangdong, China; 4 Environmental and Occupational Health Sciences Institute (EOHSI), Rutgers University, Piscataway, New Jersey; 5 VA New Jersey Health Care System, Veterans Administration Medical Center, East Orange, New Jersey

**Keywords:** FGF15, Bile acid, Drug-metabolizing enzymes, Growth hormone, Sexual dimorphism

## Abstract

Hepatic sexual dimorphism is critical for maintaining sex-specific metabolic pathways and xenobiotic clearance. We have demonstrated that in fibroblast growth factor 15 (*Fgf15*) transgenic (Tg) mice, FGF15 overexpression significantly reduced bile acid (BA) levels and induced hepatic feminization in males, marked by a male-to-female shift in expression of the drug metabolizing enzymes. This feminization is linked to the disruption of pulsatile growth hormone (GH) secretion pattern and signaling. However, it remained unclear whether FGF15 overexpression broadly disrupts hepatic sexual dimorphism or specifically drives a directional feminization program independent of biological sex. In this study, we profiled the hepatic transcriptome of female *Fgf15* Tg mice and replenished male *Fgf15* Tg mice with exogenous, pulsatile GH to test to what degree changes in the GH axis maintain liver dimorphism under low BA conditions. Beyond suppressing BA synthesis and altering steroid metabolism, female Tg mice altered expression of genes in xenobiotic metabolism and detoxification pathways, suggesting that the FGF15/BA axis regulates xenobiotic processing in females as well. Notably, FGF15 overexpression further amplified the baseline female expression profile of drug-metabolizing enzymes, characterized by the upregulation of female-predominant genes (*Cyp2b9*, *Cyp3a41*, *Sult1e1*, and *Ugt1a1*) and the suppression of male-predominant genes (*Cyp7b1*, *Cyp2d9*, *Hsd3b5*, and *Igf1*). Furthermore, pulsatile GH replenishment in male Tg mice partially restored male-predominant gene expression patterns. Taken together, these findings demonstrate that overexpression of intestine-derived FGF15 drives the feminization of sexually dimorphic hepatic gene expression independent of biological sex, a process that is at least partially mediated by impaired GH signaling.

**Significance Statement:**

Fibroblast growth factor 15 (*Fgf15*) overexpression intensified hepatic feminization of drug-metabolizing enzyme expression in female *Fgf15* transgenic mice, similar to male *Fgf15* transgenic mice. Restoring growth hormone signaling partially rescued the male-predominant gene expression pattern in male *Fgf15* transgenic mice. This indicates that disruptions to growth hormone pulsatility, alongside sustained hepatic signal transducer and activator of transcription 5 activation, contribute to FGF15–induced hepatic gene feminization.

## 1 Introduction

The liver exhibits pronounced sexual dimorphism in drug metabolism and pharmacokinetics, contributing to the variability between females and males in the metabolism of many drugs and xenobiotics.^1^ Sex-specific expression of hepatic drug metabolizing enzymes (DMEs), including phase I cytochrome P450 enzymes, and phase II sulfotransferases, glutathione transferases, and UDP-glucuronosyltransferases, metabolizes fatty acids, steroids, drugs, and environmental chemicals through phase I (oxidation) and phase II (conjugation) reactions,^2^ and causes significant variability in sex-specific drug responses and adverse drug reactions. These sex differences are driven by multiple factors, including sex hormones, growth hormone (GH) secretion patterns, aging, and circadian rhythms. Moreover, sexual dimorphism in liver gene expression extends beyond DMEs and affects at least a thousand genes in rodents, highlighting that maintaining sex-specific hepatic characteristics is essential for proper liver function.^3^

GH is primarily secreted by the pituitary gland, and one of its main functions is to regulate the expression of genes involved in the metabolism of macronutrients, xenobiotics, and steroid hormones.^4^ The sex difference in pituitary GH secretion levels and pattern is largely responsible for the sex-biased expression pattern of hundreds of genes, which controls the sexual dimorphism in liver physiology and pathology.^5^, ^6^, ^7^, ^8^, ^9^ Specifically, in males, GH is released in intermittent pulses with peak release of high levels of GH, whereas in females, GH secretion is low and nearly continuous.^10^ In the liver, GH binds to its receptor and activates the Janus kinase 2 (JAK2)-signal transducer and activator of transcription 5 (STAT5) pathway, with STAT5b, the predominant liver isoform, undergoing dimerization, nuclear translocation, and regulation of target genes.^11^^,^^12^ Consequently, STAT5b activity in the male liver is pulsatile, closely following GH pulses, whereas in the female liver, STAT5b remains persistently active.^13^^,^^14^ Continuous GH infusion in male mice feminizes hepatic gene expression by replacing the male pulsatile GH pattern with a persistent female-like signal, repressing male-biased genes and inducing female-biased genes via both STAT5-dependent and independent pathways.^15^^,^^16^

Fibroblast growth factor 15 (FGF15, human ortholog fibroblast growth factor 19, [FGF19]) is made in ileal enterocytes and functions as an endocrine hormone by suppressing hepatic bile acid (BA) synthesis.^17^^,^^18^ BAs are undergoing enterohepatic circulation, with most of them reabsorbed in the ileum, where BAs activate the farnesoid X receptor (FXR) to induce FGF15/19. Through portal circulation, FGF15/19 goes to the liver to initiate a negative feedback suppression of BA synthesis.^17^, ^18^, ^19^, ^20^ Specifically, FGF15/19 activates fibroblast growth factor receptor 4 (FGFR4) on hepatocytes and leads to the activation of the mitogen-activated protein kinase signaling pathways, including extracellular signal-regulated kinase and c-Jun N-terminal kinase, to reduce BA production by suppressing the expression of genes involved in the classical pathway of BA synthesis, including *CYP7A1/Cyp7a1* and *CYP8B1/Cyp8b1*.^17^, ^18^, ^19^, ^20^ Our previous studies demonstrated that overexpression of FGF15 (*Fgf15* transgenic [Tg] mice) markedly reduces the BA pool,^21^^,^^22^ and results in a significant change in hepatic sexual dimorphism in male mice,^21^ primarily characterized by the feminization of the male hepatic DME profile. This includes a significant upregulation of female-predominant genes (*Cyp2b9*, *Cyp2b13*, *Cyp17a1*, *Sult1e1*, *Hsd3b1*, etc), along with the downregulation of male-predominant genes (*Hsd3b5*, *Ugt2b1*, *Slco1a1*, *Mup1*, *Cyp2d9*, etc^21^). This hepatic feminization in male Tg mice is strongly associated with disrupted GH signaling.^21^ However, serum GH levels in female mice remained consistently low, with no significant differences observed between the wild-type (WT) and FGF15-Tg mice.^21^ Therefore, it remained unclear whether FGF15 overexpression broadly disrupts hepatic sexual dimorphism or specifically drives a directional feminization program independent of biological sex. Moreover, although altered GH pulsatility was implicated, the extent to which the GH signaling alone accounts for this feminized hepatic phenotype remained unknown.

In this study, we further elucidated the role of the GH-STAT5 axis in FGF15-induced liver feminization by characterizing hepatic DME expression profiles in female Tg mice and subsequently evaluating the effects of pulsatile GH replenishment on sexually dimorphic DME gene expression in male Tg mice.

## 2 Materials and methods

### 2.1 Animal and treatment

Age-matched male and female WT and *Fgf15* Tg mice on the C57BL/6J genetic background were used in all experiments. The Tg mouse model was generated using a Tet-Off system, with constitutive overexpression of *Fgf15* in both the liver and intestine.^22^ All animal experiments were performed in accordance with protocols approved by the Institutional Animal Care and Use Committee at Rutgers University.

Recombinant human growth hormone (hGH) was purchased from Genscript Biotech (Cat # Z03012). The dose and injection schedule were referred to previous publications^23^ and validated by our pilot experiment. In the pilot experiment, age-matched female WT and Tg mice were used to test hGH biological activities. Mice were administered a single intraperitoneal injection of either 0.1% bovine serum albumin (Veh), 0.2 mg/kg, or 1 mg/kg hGH. At 0, 1, 2, 3, and 4 hours postinjection, 20 *μ*L of blood was collected from the tail vein of each mouse, and the hGH level in the serum was determined by a hGH ELISA kit (Thermo Fisher Scientific, #EHGH1). To determine the required sample size for the GH replenishment experiment, a power analysis was conducted using the GH-STAT5b activation metrics derived from the pilot study. The analysis indicated that a cohort size of *n* = 4/group could provide sufficient statistical power to characterize the pharmacokinetic profile of circulating hGH postinjection. To maximize statistical power and account for potential technical or biological complications, additional mice were proactively enrolled in each experimental group.

For the GH replenishment study, 6-month-old male WT and Tg mice were assigned to 4 experimental groups: WT/Veh, WT/GH, Tg/Veh, and Tg/GH. To mimic the characteristic male pulsatile pattern, mice received intraperitoneal injections twice daily for 3 days of either 0.1% bovine serum albumin (Veh) or hGH (0.5 mg/kg). Serum and liver tissue were harvested 2 hours after the final injection. To minimize circadian rhythm variations, all tissue collections were restricted to a window between 10 am and 12 pm. Collected samples were subsequently analyzed to evaluate hepatic GH-STAT5 signaling and downstream gene expression levels. Serum insulin-like growth factor 1 (IGF-1) levels were quantified using the mouse IGF-1 quantikine ELISA kit from R&D Systems.

### 2.2 RNA-sequencing analysis

Total RNA was isolated from frozen liver tissue of female WT and Tg mice using TRIzol reagent (Thermo Fisher Scientific). Transcriptome sequencing service (mRNA sequencing) and subsequent bioinformatic analyses, including differential expression (DE) analysis and functional enrichment analysis (principal component analysis, volcano plots, and gene ontology (GO) enrichment), were performed by Admera Health. Briefly, DE analysis was conducted using DESeq2, and *P* values were adjusted using multiple testing using the Benjamini-Hochberg–false discovery rate method. Genes demonstrating a fold change ≥1.5 and an adjusted *P* value (*p*_*adj*_) <.05 were considered significantly differentially expressed.

### 2.3 Gene expression

Relative gene expression was determined as previously described.^24^ Briefly, total RNA was extracted from frozen liver or ileal tissues using TRIzol reagent. cDNA was synthesized by reverse transcription. Quantitative real-time polymerase chain reaction (PCR) was performed using SYBR Green on the Applied Biosystems ViiA7 Real-Time PCR System (Life Technologies). Relative gene expression levels were calculated using the 2ˆ^-ΔΔCt^ method and normalized to *β*-actin. Primer sequences used in this study are listed in Supplemental Table 1.

### 2.4 Western blot analysis

Liver tissues were homogenized and lysed in radioimmunoprecipitation assay buffer supplemented with protease and phosphatase inhibitors (Thermo Fisher Scientific). Protein samples (20 *μ*g per lane) were separated by 10% SDS-PAGE and transferred to polyvinylidene difluoride membranes. Membranes were blocked with 5% nonfat milk for 2 hours at room temperature. The corresponding primary antibodies against mouse phosphorylated STAT5 (p-STAT5) (Y694) (Cell Signaling Technology, CST #4322, 1:1000), STAT5 A/B (R&D Systems, #AF2168, 1:1000), and *β*-actin (CST #3700, 1:2000) were diluted to the recommended dilution with 5% nonfat milk. After blocking, the membrane was incubated with the corresponding primary antibodies at 4 °C overnight with gentle shaking and was then washed before being incubated with the corresponding secondary antibody at a dilution of 1:10,000 for 1 hour at room temperature. Protein bands were visualized using enhanced chemiluminescence substrates (Thermo Fisher Scientific). Following detection of the target protein, the membrane was stripped using mild stripping buffer (0.2 M glycine, pH 2.2, 1% Tween-20, 0.1% SDS) and reprobed with the subsequent antibody. *β*-actin from the same membrane served as the internal loading control for normalization. Equal amounts of total protein for each sample were loaded across separate gels, and the band intensities of the target protein bands were quantified using ImageJ software (https://imagej.nih.gov/ij/).

### 2.5 Statistical analysis for quantitative PCR and western blot data

Data are expressed as mean ± SD. Statistical differences among multiple groups were evaluated using one-way ANOVA followed by Tukey’s post hoc test, unless specified otherwise. For datasets that violated the assumptions of parametric testing, the nonparametric Kruskal-Wallis test was employed. All statistical analyses were performed using SAS Studio (SAS Institute Inc). A *P* value <.05 was considered statistically significant.

## 3 Results

### 3.1 Differentially expressed genes in the livers of female Tg mice cluster into metabolic pathways

Principal component analysis (PCA) of RNA-sequencing data showed a clear separation between female WT and Tg mice, and confirmed that biological replicates clustered within each group, indicating high reproducibility and global transcriptional differences between genotypes (Fig. 1A). Differential expression analysis (DESeq2, fold change ≥1.5, *P* (*p*_*adj*_) <.05, Fig. 1B) identified 376 genes altered in female Tg mice compared with WT mice, with 111 upregulated and 266 downregulated (Supplemental Table 2). Kyoto Encyclopedia of Genes and Genomes pathway enrichment analysis revealed that these differentially expressed genes were significantly enriched in pathways related to estrogen signaling, steroid hormone biosynthesis, xenobiotic metabolism, retinol metabolism, bile secretion, and GH synthesis (Fig. 1C, adjusted *P* < .05). These results demonstrated that FGF15 overexpression markedly remodeled hepatic metabolic gene expression in female mice, thereby reshaping the hepatic sexual dimorphism and sex-biased pathways.

**Fig. 1:**
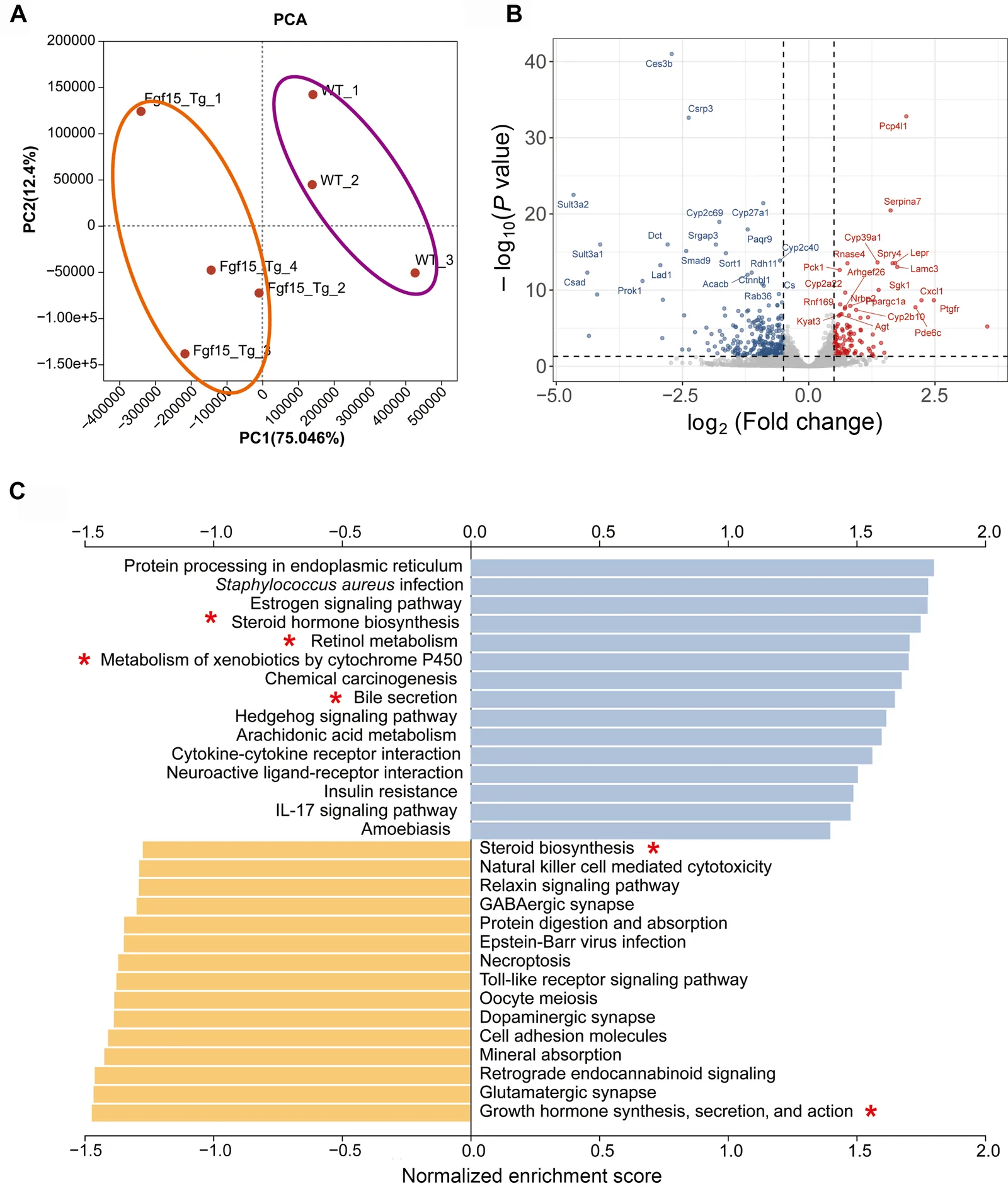
Genes with altered expression in female *Fgf15* Tg mouse livers cluster into metabolic pathways. (A) Principal component analysis (PCA) of RNA-sequencing data shows clear separation between female WT and *Fgf15* Tg mice. (B) Volcano plot of differentially expressed genes between female WT and Tg mice. Red dots indicate upregulated genes; blue dots indicate downregulated genes (fold change > 1.5, *P* (*p*_*adj*_) <.05). (C) KEGG pathway enrichment analysis of differentially expressed genes. The bar plot shows the top enriched pathways. n = 3 and 4 for WT and Tg, respectively. IL, interleukin; KEGG, Kyoto Encyclopedia of Genes and Genomes.

### 3.2 Alterations in hepatic DME gene expression in female Tg mice

To understand the impact of FGF15 overexpression on hepatic DME gene expression, key metabolic pathways were further analyzed. In the xenobiotic metabolism pathway, phase II DME genes *Sult2a3*, *Sult2a4*, and *Gstt3* were significantly upregulated, indicating the activation of detoxification processes (Fig. 2A). In the steroid hormone biosynthesis pathway, representative genes, including *Sult1e1*, *Cyp2b10*, *Cyp17a1*, *Cyp3a25*, and *Cyp2b9*, were significantly upregulated in female Tg mice (Fig. 2B), suggesting enhanced female-biased steroid metabolism. For BA metabolism and secretion, *Sult2a3*, *Sult2a4*, *Abcc4*, and *Slco1a4* showed elevated expression (Fig. 2C**)**, suggesting increased BA detoxification and sinusoidal efflux, whereas for retinol metabolism, steroidogenesis, and xenobiotic detoxification, *Cyp4a31*, *Cyp26b1*, *Cyp2b13*, *Cyp2a5*, and *Rdh9* were upregulated, consistent with enhanced female-biased metabolic activity (Fig. 2D). Conversely, genes involved in GH signaling, including *Stat1*, *Adcy10*, *Igfals*, and *Igf1*, were downregulated in female Tg mice, reflecting a potential disruption of the GH-STAT5b signaling. Collectively, these results indicate that FGF15 overexpression in female mice further enhances female-predominant hepatic metabolic pathways, such as xenobiotic metabolism, hormone clearance, and detoxification, while suppressing male-predominant pathways, including GH signaling. This hyperfeminization of the female liver transcriptome aligns with the partial hepatic feminization previously observed in male Tg mice.

**Fig. 2:**
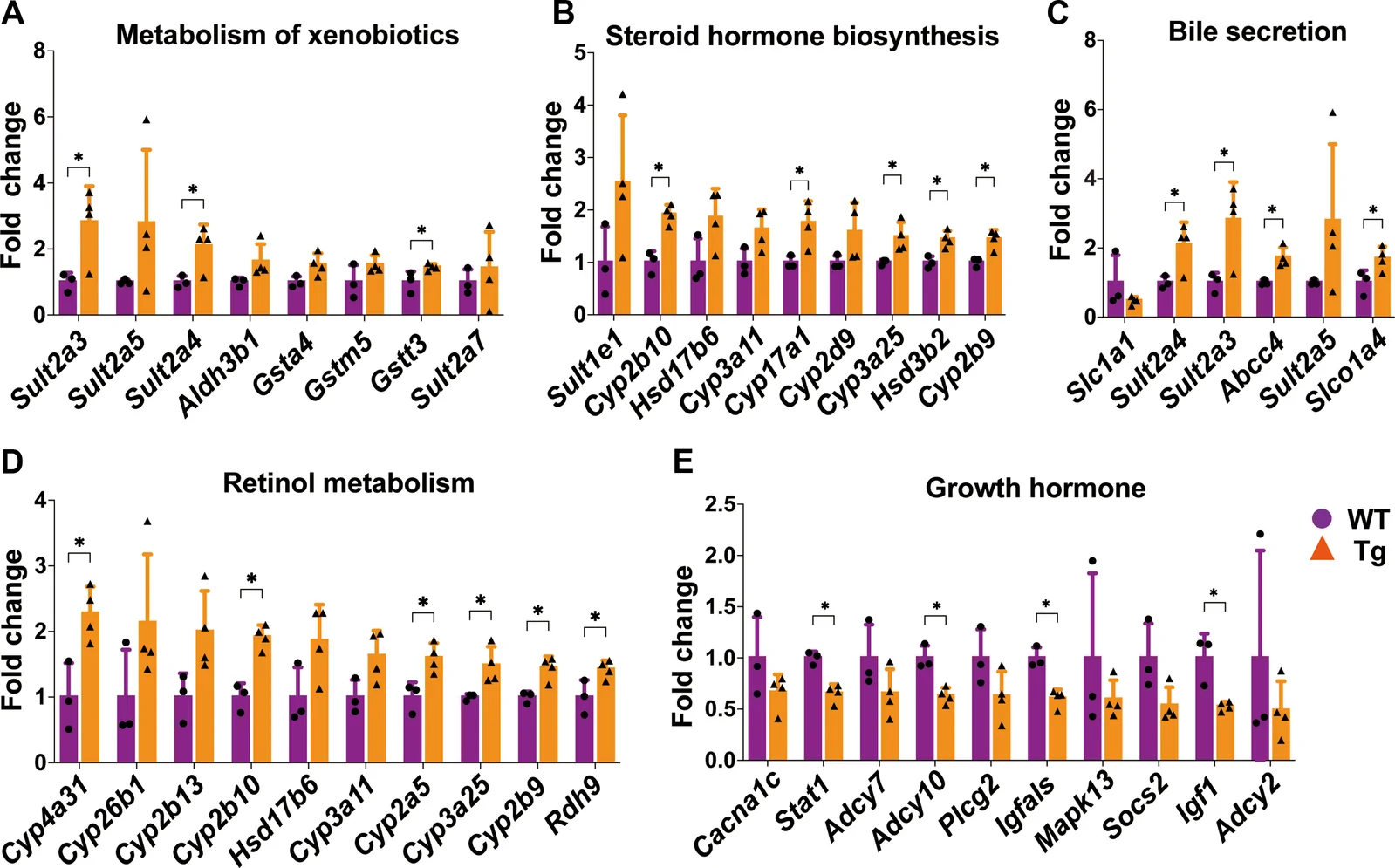
Enhanced feminization of hepatic DME gene expression in female *Fgf15* Tg mice. (A) Altered expression of genes involved in steroid hormone biosynthesis in female *Fgf15* Tg mice. (B) Altered expression of genes involved in the metabolism of xenobiotics by cytochrome P450. (C) Altered expression of genes involved in bile secretion. (D) Altered expression of genes involved in retinol metabolism. (E) Altered expression of genes involved in GH synthesis. *n* = 3 and 4 for WT and Tg mice, respectively. ∗*P* < .05 between the indicated groups.

Female-predominant hepatic genes, including *Cyp2b9*, *Cyp2b10*, *Sult2a4*, *Cyp39a1*, and *Cyp2c29*, were significantly upregulated in female Tg mice (Fig. 3A), whereas male-predominant genes, such as *Igf1*, *Bcl6*, and *Elovl3,* were markedly downregulated (Fig. 3B). Consistent with our previous findings in male Tg mice, FGF15 overexpression in female mice also promoted a feminized hepatic gene expression pattern, characterized by upregulation of female-biased genes and downregulation of male-biased genes.^21^ Moreover, protein analysis showed that female mice maintained a sustained pattern of p-STAT5 activation, which was further enhanced by FGF15 overexpression (Fig. 3C). This suggests that FGF15 overexpression may shift the hepatic transcriptional profile toward a female-like pattern in both sexes but induce pronounced feminizing effects in male mice.

**Fig. 3:**
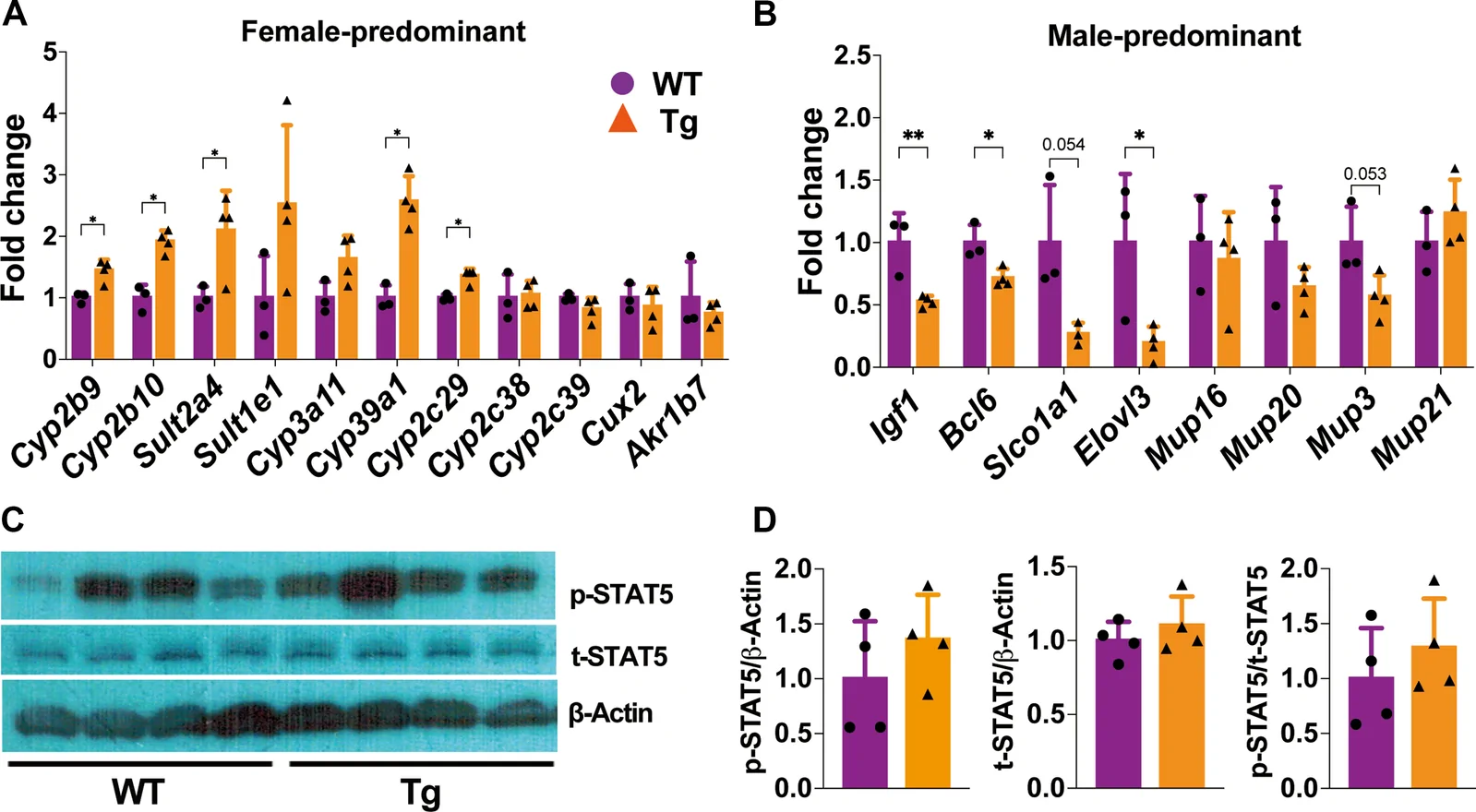
Upregulation of female-predominant genes but downregulation of male-predominant genes in female *Fgf15* Tg mice. (A) Altered expression of genes involved in female-predominant liver genes in female Tg mice. (B) Altered expression of genes involved in male-predominant liver genes in female Tg mice. *n* = 3 and 4 for WT and Tg mice, respectively. (C) Western blot analysis and (D) quantification of protein levels in the STAT5 signaling pathway in the female mouse livers, with protein levels quantified by densitometry, and the ratio of p- to total protein (t-) was used to indicate activation of the STAT5 signaling pathway. ∗*P* < .05, ∗∗*P* < .01 between the indicated groups.

### 3.3 Pulsatile GH supplementation restores the male pattern of STAT5 activation

A pilot study was performed to clarify whether recombinant hGH enters systemic circulation and activates hepatic STAT5 signaling in mice after intraperitoneal administration. Serum hGH kinetics were profiled over 4 hours (Fig. 4A). Following injection, serum hGH levels increased dose dependently and peaked at 1 hour with the 0.2 mg/kg dose and at 2 hours with the 1.0 mg/kg dose. By 3 hours postinjection, serum hGH levels returned to nearly undetectable baseline levels in both groups (Fig. 4A). Following the optimization of hGH dosing for STAT5 activation, male Tg mice were given twice daily exogenous hGH injections to simulate the pulsatile pattern of male GH secretion (Fig. 4B). Hepatic p-STAT5 levels in WT mice exhibited marked variability, displaying a mixture of high and low activation states across individual animals. This fluctuation is consistent with the distinct peaks and troughs characteristic of pulsatile GH activation in males.^25^ In contrast, male Tg mice lacked this pulsatile variability, presenting lower but constant p-STAT5 levels **(**Fig. 4B), a pattern consistent with our previous observations.^21^ After chronic pulsatile hGH administration, hepatic p-STAT5 levels were upregulated in both WT and Tg mice (Fig. 4, B and C). However, no significant differences were detected across strains, as demonstrated by a comparable p-STAT5 to total protein-STAT5 ratio (Fig. 4, B and C). Nevertheless, Tg mice showed a stronger response to exogenous GH stimulation than WT controls, given their lower baseline p-STAT5 levels **(**Fig. 4C). Because IGF-1 is primarily synthesized by the liver under the control of GH signaling, serum IGF-1 levels were quantified next. Circulating IGF-1 levels were significantly reduced in Tg mice (Fig. 4D), reflecting their suppressed GH signaling **(**Fig. 4B). However, following exogenous GH administration, serum IGF-1 levels did not correlate with the degree of p-STAT5 activation in these mice (Fig. 4D).

**Fig. 4:**
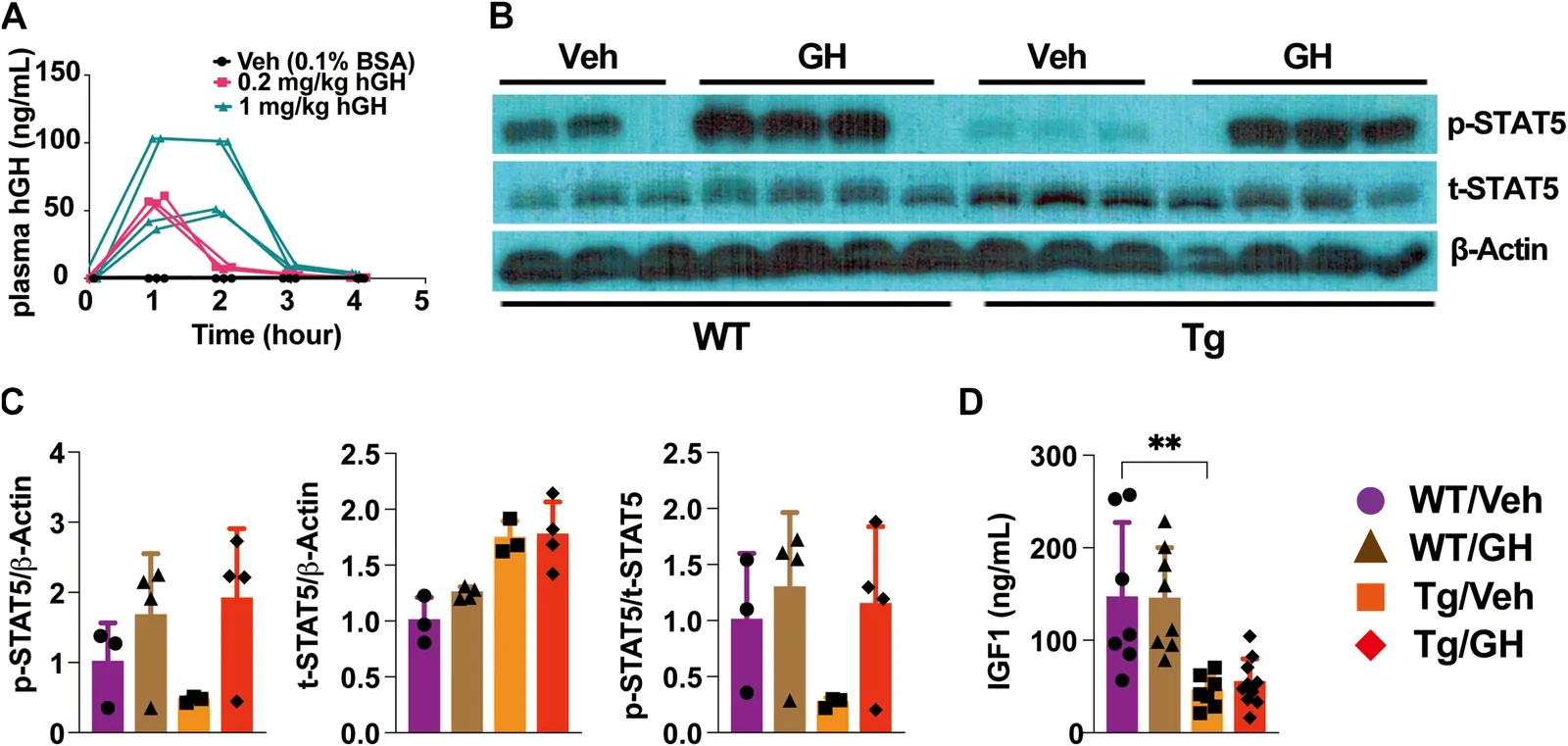
Pulsatile GH supplementation restored the male pattern of STAT5 activation in male *Fgf15* Tg mice. (A) Serum hGH levels after intraperitoneal administration of exogenous recombinant hGH. The levels of hGH display time-course dynamics, peaking at 1 hour for a 0.2 mg/kg dose or 1 to 2 hours for a 1 mg/kg dose, before gradually returning to baseline within 4 hours. (B) Activation of the STAT5 signaling pathway after pulsed GH supplementation in male mice. (C) The protein levels were quantified by densitometry, and the ratio of p-STAT5 to t-STAT5 was used as an indication of the activation of the STAT5 signaling pathway. (D) Serum IGF-1 measured by ELISA after pulsed hGH supplementation in male mice (*n* = 7, 8, 7, and 10/group for WT/Veh, WT/GH, Tg/Veh, and Tg/GH, respectively). ∗∗*P* < .01 between the indicated groups.

### 3.4 Pulsatile STAT5 activation partially restores male-predominant liver DME expression pattern in male Tg mice

Among hepatic DME genes, the expression level of class I male-predominant genes in male Tg mice was significantly downregulated compared with male WT controls, to approximately 60% (*Cyp2d9*), 50% (*Hsd3b5*), 60% (*Slco1a1*), and 10% (*Igf1*) of WT levels (Fig. 5A). Similarly, class II male-predominant genes were also significantly downregulated to 50% (*Cyp7b1*), 40% (*Srd5a1*), 10% (*Cyp7a1*), and 80% (*Igfals*) of the levels observed in male WT mice (Fig. 5B). Following pulsatile GH administration, both class I and class II male-predominant genes were upregulated in both strains. Except for *Hsd3b5*, which showed stronger induction in WT mice, all these genes tended to show a stronger upregulation in Tg mice (Fig. 5, A and B).

**Fig. 5:**
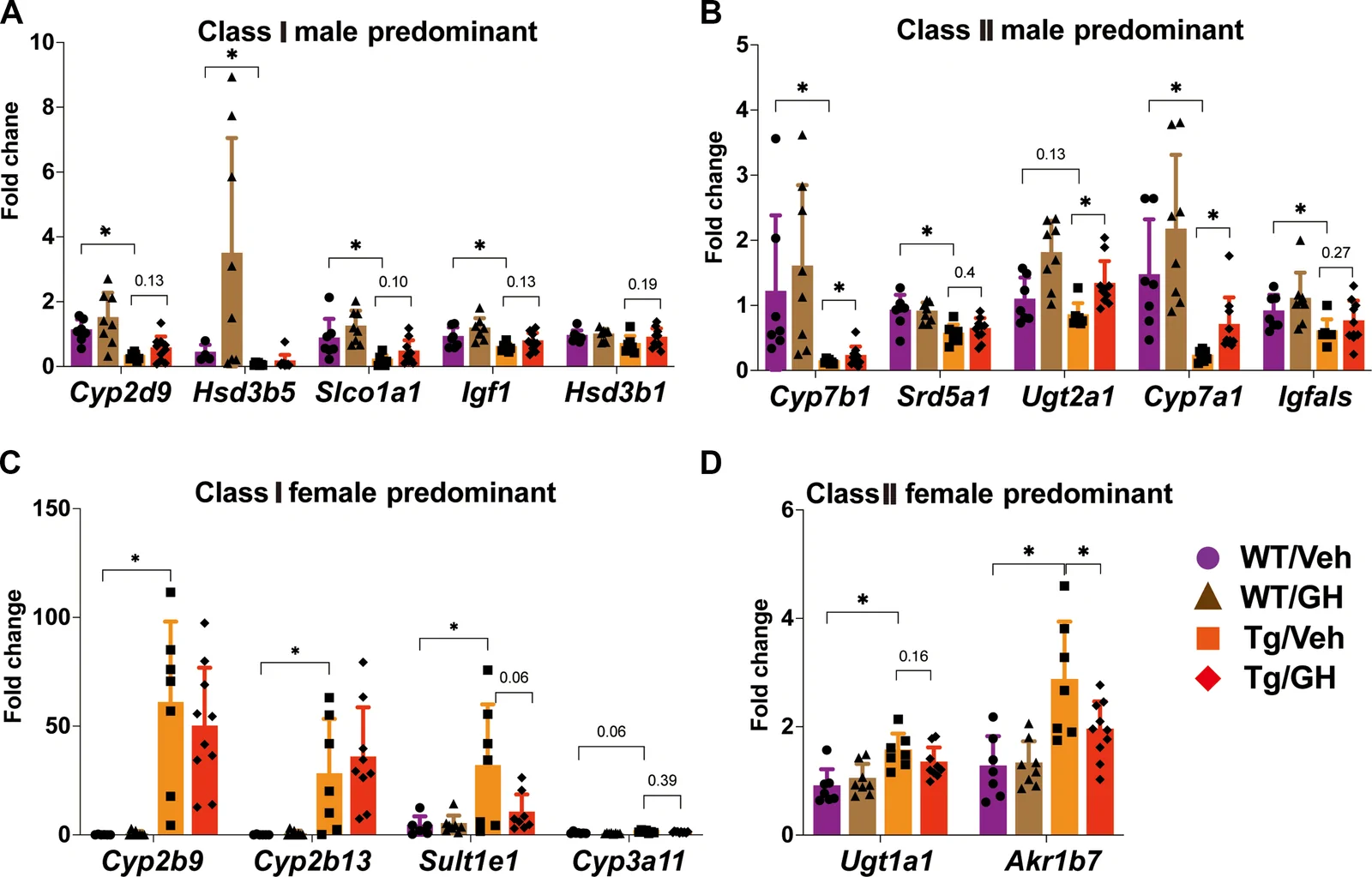
Pulsatile STAT5 activation by hGH partially restored male-predominant liver DME gene expression pattern in male *Fgf15* Tg mice. Expression levels of representative (A) class I and (B) class II male-predominant liver genes, as well as representative (C) class I and (D) class II female-predominant liver genes following hGH supplementation in male WT and Tg mice (*n* = 7, 8, 7, and 10 for WT/Veh, WT/GH, Tg/Veh, and Tg/GH, respectively). ∗*P* < .05 between the indicated groups.< 0.05 between the indicated groups.

*Cyp2b9*, *Cyp2b13*, and *Sult1e1* are class I female-predominant genes. Compared with WT mice, their expression level in Tg mice was significantly increased by more than 50-, 20-, and 20-fold, respectively (Fig. 5C). Regarding class II female-predominant genes, *Sult1a1* expression increased 1.8-fold and *Akr1b7* expression increased 3-fold in Tg mice compared with WT mice (Fig. 5D). Interestingly, after pulsatile GH administration, these female-predominant genes, including *Cyp2b9*, *Sult1e1*, *Ugt1a1*, and *Akr1b7*, were downregulated in Tg mice but remained unchanged in WT mice (Fig. 5, C and D). Taken together, these results demonstrated that pulsatile GH supplementation partially rescues the feminized expression profile of hepatic metabolic genes and helps to restore the native sexual dimorphism in male Tg mice.

## 4 Discussion

The regulation of sexually dimorphic liver metabolism and drug metabolism by GH signaling has been extensively studied for several decades.^15^^,^^25^^,^^26^ These metabolic dimorphisms are driven by sex-specific pituitary GH secretory profiles. In males, GH secretion features high-amplitude, low-frequency pulses that cause intermittent, pulsatile activation of hepatic STAT5b.^13^^,^^14^ Conversely, the female GH profile is characterized by high-frequency, low-amplitude pulses that maintain continuous STAT5b activation (Fig. 6A). Our previous study found that FGF15 overexpression in male mice induced a feminized hepatic gene expression pattern associated with reduced GH signaling and disturbed GH pulsatility,^21^ and this study further demonstrated that FGF15 overexpression enhances female-predominant hepatic gene expression in female Tg mice, indicating that overexpressed FGF15 promotes hepatic feminization independent of sex. Restoring pulsatile STAT5 activation in males in this study only partially reversed the feminine DME expression pattern, implying that alternative regulatory pathways contribute to FGF15-mediated hepatic dimorphism. Taken together, we hypothesize a working model in which FGF15 overexpression promotes the hepatic feminization of DME expression (Fig. 6B). In this hypothetical model, FGF15 overexpression suppresses BA synthesis and reduces systemic BA levels, which might interfere with the endogenous pulsatile GH secretion pattern of males. This disrupted GH pattern subsequently shifts hepatic STAT5 signaling from a male-like intermittent activation toward a more sustained activation pattern, thereby driving feminized liver DME expression and altered metabolic function. Furthermore, hepatic FGF15/FGFR4 signaling may also crosstalk with and modulate STAT5 activity. However, these hypothetical mechanisms require further determination in the future.

**Fig. 6:**
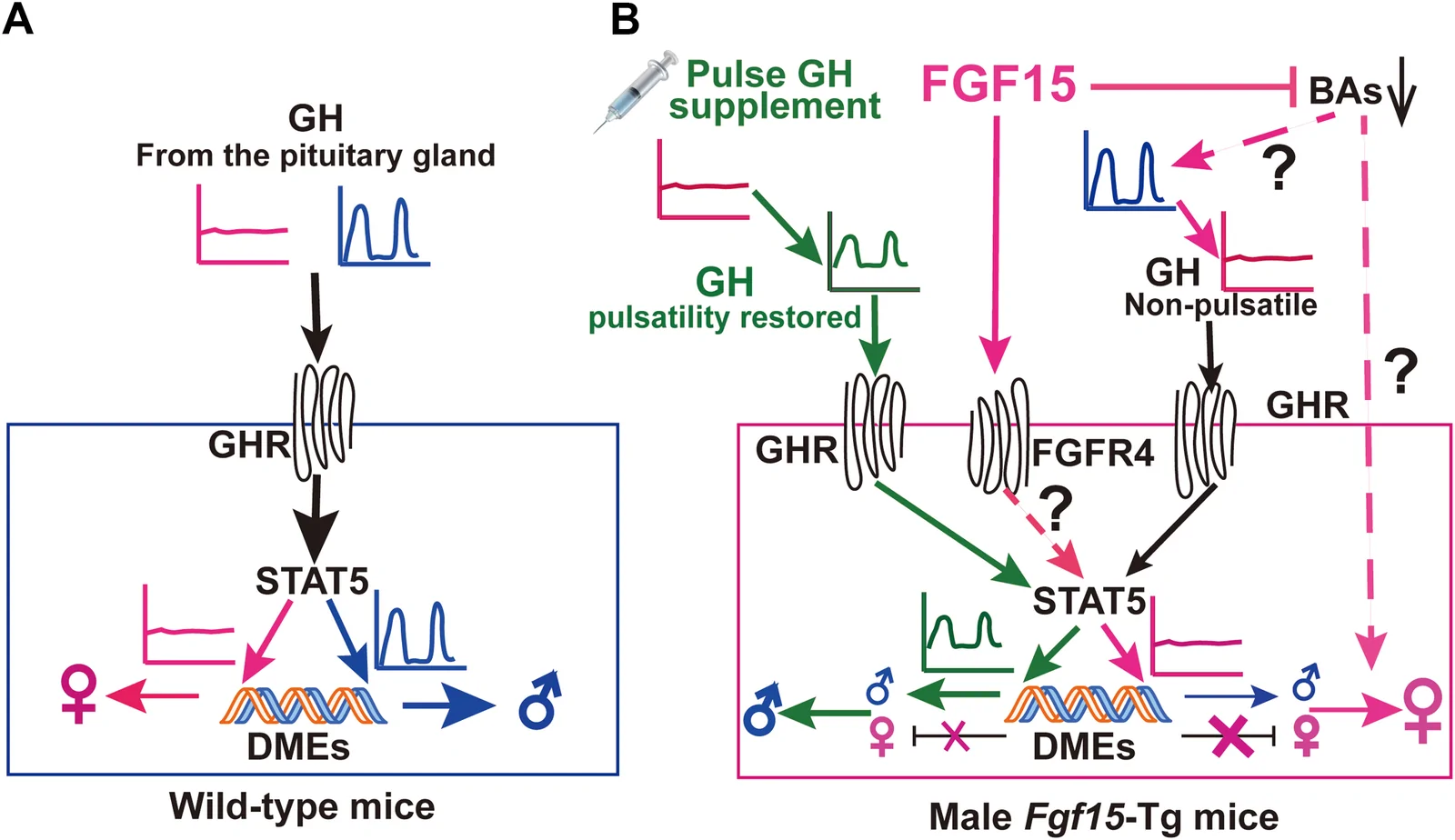
Schematic diagram of possible mechanisms leading to alteration in sexual dimorphic expression of hepatic DMEs in *Fgf15* Tg mice. (A) In WT mice, female GH secretion from the pituitary gland exhibits a continuous GH release at low levels, whereas male GH secretion shows a pulsatile GH-releasing pattern with high GH levels at peak. This distinct sexual dimorphic pattern of GH secretion regulates sex-specific hepatic gene expression of DMEs. (B) This is the proposed hypothetical model showing that FGF15 overexpression in Tg mice activates its receptor FGFR4 and downstream signaling cascades, which suppresses BA synthesis, thereby reducing BA levels. Reduced BA levels and/or signaling, in combination with activated FGFR4, might interact with the GH-STAT5 axis, which is hypothesized to shift hepatic STAT5 signaling from a male pulsatile activation pattern to a female sustained activation pattern, thereby driving a switch in liver sexual dimorphism of gene expression. The molecular mechanisms underlying this transition in *Fgf15* Tg mice require further elucidation.

As signaling molecules, BAs regulate lipid metabolism and energy expenditure largely through the activation of nuclear and membrane receptor pathways, including FXR, TGR5, PXR, and S1PR2,^27^ so BA pool size is strictly controlled via the intestinal FXR-FGF15/19 feedback loop.^17^^,^^18^ BA levels and BA composition, as well as FXR activation, exhibit sex-dependent differences, which contribute to sex differences in cholesterol, lipid, and xenobiotic metabolism.^28^, ^29^, ^30^ In Tg mice, reduced total BA levels or pool size may therefore contribute, at least in part, to altered hepatic sexual dimorphism. However, a direct FGFR4-STAT5 pathway in hepatocytes has not been established. Furthermore, FGF15-FGFR4 signaling may influence hepatic sex-biased gene expression through additional mechanisms that require further investigation.

Because FGF15 serves as a functional ortholog to human FGF19, these findings reveal critical translational considerations for engineered FGF19 biologics and the chronic clinical administration of FXR agonists. FGF19-based therapeutics, such as aldafermin (NGM282), have been developed to treat metabolic and cholestatic liver diseases,^31^ whereas FGFR4 inhibitors, such as fisogatinib (BLU-554), are clinically investigated to target FGF19/FGFR4-driven hepatocellular carcinoma.^32^ Pharmacological modulation of the FGF19-FGFR4 axis could fundamentally disrupt individual pharmacokinetics, triggering systemic alterations in drug clearance and elevating the risk of drug-drug interactions. Although future studies need to determine whether this regulatory mechanism is conserved in humans, our data highlight a novel role for the FXR-FGF15/19 axis and BA homeostasis in modulating hepatic sexual dimorphism of DMEs.

Despite the novel insights presented in this study, further investigations are required to address the limitations and validate these mechanisms. Although we restored pulsatile GH signaling and STAT5 phosphorylation in male Tg mice successfully by replenishing male Tg mice with hGH, the feminization of hepatic DME gene expression was only partially reversed. This partial rescue suggests that optimizing the GH dosing schedule in the future may be necessary, or that alternative mechanisms independent of GH signaling may also contribute to FGF15-induced hepatic feminization. Similar findings have been reported in previous studies, in which pulsatile GH replacement (administered via intraperitoneal injection at 12-hour intervals for 7 days) restored male pattern liver gene expression and physiological responses in hypophysectomized mice through a STAT5b-dependent mechanism.^13^^,^^23^^,^^26^^,^^33^ Our 3-day GH protocol may not optimally simulate the endogenous male GH secretory patterns, and thus it is not sufficient to match the precise pulse amplitude, interpulse intervals, and baseline troughs required to sustain male-predominant hepatic profiles.^8^^,^^23^^,^^34^ Moreover, previous studies indicate that although acute GH pulses rapidly activate hepatic STAT5, the full restoration of sex-biased hepatic gene expression often requires sustained or long-term hormonal stimulation, likely involving secondary transcriptional cascades and chromatin remodeling events.^33^^,^^35^ Alternatively, the incomplete rescue observed here may result from parallel, non-GH mechanisms. Given that FGF15/19 can directly regulate hepatic metabolic gene expression through cascades such as cAMP response element-binding protein (CREB)-PPARGC1A gene PPARG coactivator 1 alpha (PGC-1α) and small heterodimer partner (SHP)-DNA methyltransferase 3A (DNMT3A),^36^^,^^37^ it is possible that FGF15 affects chromatin modifications, permanently altering the hepatic epigenetic landscape and rendering certain female-predominant genes resistant to acute GH restoration.

Another consideration in this study is that serum IGF-1 levels did not directly parallel hepatic p-STAT5 activation after GH stimulation. IGF-1 is primarily produced in the liver, which accounts for most of the circulating IGF-1. Following transcription and alternative splicing of the *Igf1* gene, the initial translation product, pre-pro-IGF-1, undergoes extensive posttranslational modification and processing (reviewed in a study by Oberbauer^38^). In addition, unbound IGF-1 is highly unstable, exhibiting a half-life of only 10–12 minutes prior to renal clearance.^39^^,^^40^ Moreover, the STAT5-suppressor of cytokine signaling 2 (SOCS2) negative feedback loop serves as a crucial molecular switch that regulates GH signaling in the liver. Upon GH-growth hormone receptor (GHR)-JAK2 activation, p-STAT5 forms homodimers that translocate into the nucleus to bind the promoters of *Igf1* and *Socs2* to initiate their transcription. As SOCS2 protein levels subsequently accumulate in the cytoplasm, they bind directly to the active GHR-JAK2 complex, shutting down the signaling cascade.^41^ Consequently, identifying and validating robust downstream biological indicators of GH signaling is a critical priority for future studies.

As observed in STAT5b-deficient or GH pulse-altered mice, disruption of the sexual dimorphism leads to loss of male- or female-biased gene expression, altered DME profiles, impaired xenobiotic metabolism, and increased susceptibility to liver disease.^42^ Our current study demonstrates that GH signaling is involved in FGF15-mediated modulation of hepatic sexual dimorphism, as indicated by changes in DME gene expression. However, measuring only DME mRNA expression risks oversimplifies the physiological reality, given that gene transcript levels often diverge from actual in vivo enzymatic activity and rate of drug metabolism. Therefore, future studies assessing DME protein levels, enzymatic activity, and in vivo pharmacokinetics are required to fully elucidate the functional impact of FGF15-induced changes on hepatic DME expression.

## Conflict of interest

The authors declare no conflicts of interest.
