# Supplementary material for: Persistent activation of growth hormone contributes to sexual dimorphic expression of hepatic drug metabolizing enzymes in Fgf15 transgenic mice
Source: Drug Metab Dispos. 2026 Jul 15;54(9):100368. doi: 10.1016/j.dmd.2026.100368 (PMC13688975; doi:10.1016/j.dmd.2026.100368)
Supplement: Supplementary file 1 [file mmc1.docx]

**Supplemental Table 1:** The primer sequences used in the Q-PCR analysis in this study.

| **Gene** | **Forward (5'→3')** | **Reverse (5'→3')** |
| --- | --- | --- |
| Akr1b7 | AAGCGGGAGGATCTCTTCAT | TCAGATCCGAGAGGGTGTTC |
| Cyp17a1 | TGGCTTTCCTGGTGCACAATC | GGAGGTGAGTCCGGTCATTGAA |
| Cyp1a2 | ACATCCTTTGTCCCCCTTCAC | GGTCTTTCCACTGCTTCTCATC |
| Cyp2b10 | GACTTTGGGATGGGAAAGAG | CCAAACACAATGGAGCAGAT |
| Cyp2b13 | GCTTTTCTACCCTTCTCCACAG | ATGTCCTTAGAAGCAACAGGG |
| CYP2B6 | CGGGGATATGGTGTGATCTT | AGTGGTCACAGAGAATCGCC |
| Cyp2b9 | TGGCCACCATGAAAGAGTTTG | GCTGTGATGCACTGGAAGAGAA |
| Cyp2c55 | ATCTTGAACTCCCCATGGATG | CACTCGCCCCAAAATGTAAC |
| Cyp2d9 | TGCAGAGATAGAGAAGGCCA | TCACGCACCACCATGAGC |
| Cyp3a11 | TCACAGACCCAGAGACGATTAAGA | CCCGCCGGTTTGTGAAG |
| Cyp3a16 | TCACCAGTGGAAATCTCAAGG | CACCTAACACATCTTTCACAGC |
| CYP3A4 | AGTGGAAAACTCAAGGAGATGG | CGATGTTCACTCCAAATGATGT |
| Cyp3a44 | ACATTCACCAGTGGAAGACTC | CCTAACACATCTTTCATAGCAACAG |
| Cyp7a1 | AACAACCTGCCAGTACTAGATAGC | GTGTAGAGTGAAGTCCTCCTTAGC |
| Fgf15 | GCCATCAAGGACGTCAGCA | CTTCCTCCGAGTAGCGAATCAG |
| Fxr | TCCGGACATTCAACCATCAC | TCACTGCACATCCCAGATCTC |
| Gsta1 | CCCCTTTCCCTCTGCTGAAG | TGCAGCTTCACTGAATCTTGAAAG |
| Gstm3 | GAAGCACAACCTGTGTGGAGA | ATCGGACTGCAGCAGACTAT |
| Hsd3b1 | ATTCCGACCAGAAACCAAGG | AGAATGTCCTTCCCAACACTG |
| Hsd3b5 | CACTAGTGAAGCAGCACAGG | AGGTTCCAGGCAATTCAGTA |
| Mup1 | AAGGTTTGCACAACTATGTGAGA | TCCTGGTGAGAAGTCTCCACTC |
| Shp | CGATCCTCTTCAACCCAGATG | AGGGCTCCAAGACTTCACACA |
| Slco1a1 | CACTGGGAGCTTTGAGATAGG | TGAGGAAATGAGGTGATGCC |
| Srd5a1 | TGTTTGCTCTGTTCACCCTG | TGGACAGCACACTAAAGCAG |
| Sult1a1 | GAAAGTGTCCTATGGGTCGTG | CGCCCCAGAAACTCTAGAATC |
| Sult1e1 | AAGGGCAAGTTCCGTATGG | CTCTCCAGGAACTCTATTAGCTTTAC |
| Ugt1a1 | GCTTCTTCCGTACCTTCTGTTG | GCTGCTGAATAACTCCAAGCAT |
| Ugt2b1 | GTGCTGGTGTGGCCTACAG | ATTGCTCGGCCCAATGAGG |
